# Offline orbitofrontal cortex reactivation depends on recency of place-reward changes and coheres with hippocampal replay

**DOI:** 10.1016/j.isci.2024.109205

**Published:** 2024-02-10

**Authors:** Silviu I. Rusu, Jeroen J. Bos, Pietro Marchesi, Jan V. Lankelma, Ildefonso Ferreira Pica, Luc J. Gentet, Marian Joëls, Cyriel Pennartz

**Affiliations:** 1 Cognitive and Systems Neuroscience Group, SILS Center for Neuroscience, University of Amsterdam, Amsterdam, the Netherlands; 2 Research Priority Program Brain and Cognition, University of Amsterdam, Amsterdam, the Netherlands; 3 Department Translational Neuroscience, University Medical Center Utrecht, Utrecht, the Netherlands; 4 School of Physiology, Pharmacology and Neuroscience, University of Bristol, Biomedical Sciences Building, University Walk, Bristol BS8 1TD, UK; 5 University of Groningen, University Medical Center Groningen, 9713 GZ Groningen, the Netherlands

**Keywords:** Neuroscience, Systems, neuroscience, Learning and memory, Sleep, Emotion and value

## Abstract

The orbitofrontal cortex, one of the key neocortical areas in valuation and emotion, is critical for cognitive flexibility but its role in the consolidation of recently acquired information remains unclear. Here, we demonstrate orbitofrontal offline replay in the context of a place-reward association task on a maze with varying goal locations. When switches in place-reward coupling were applied, replay was enhanced relative to sessions with stable contingencies. Moreover, replay strength was positively correlated with the subsequent overnight change in behavioral performance. Interrogating relationships between orbitofrontal and hippocampal activity, we found that orbitofrontal and hippocampal replay could occur independently but became coordinated during a type of cortical state with strong spiking activity. These findings reveal a structured form of offline orbitofrontal ensemble activity that is correlated with cognitive flexibility required to adapt to changing task contingencies, and becomes associated with hippocampal replay only during a specific state of high cortical excitability.

## Highlights

• Ensembles in orbitofrontal cortex show offline replay after place-reward learning
• Switches in place-reward coupling on a circular maze enhance replay strength
• Replay strength correlates positively with behavioral performance the next day
• Orbitofrontal and hippocampal cells replay together when cortex is highly active

## Graphical abstract

**Figure:**
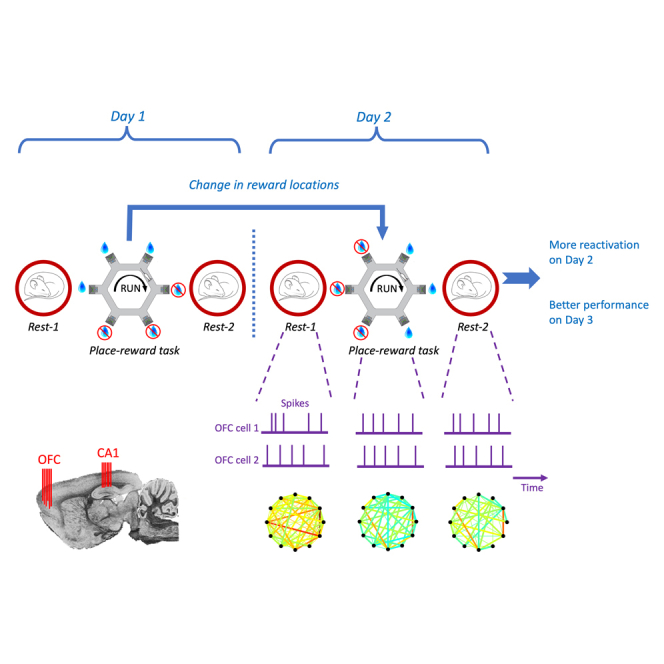

## Introduction

The orbitofrontal cortex (OFC) has been implicated in a range of cognitive functions. OFC lesions have been associated with deficits in flexibly coding stimulus-outcome associations.^1^^,^^2^^,^^3^^,^^4^^,^^5^ Alternatively, OFC functioning has been cast as signaling the economic value of choice options,^6^^,^^7^^,^^8^ signaling reward expectancy,^9^^,^^10^^,^^11^ or supporting credit assignment during multi-option tasks by encoding action-outcome associations.^12^^,^^13^ Several electrophysiological studies have previously attempted to identify the neuronal correlates of these functions. For instance, firing activity and rhythmic phase-locking of single OFC units was shown to correlate with the outcome value of specific sensory cues^9^^,^^11^^,^^14^^,^^15^ and to be sensitive to reversal learning.^11^^,^^16^^,^^17^

However, previous observations pointing to causal roles of the OFC in outcome devaluation^18^^,^^19^^,^^20^ and sensory preconditioning^21^ have been difficult to explain using only the hypotheses described previously.^22^ To account for its functional versatility, the OFC has been proposed to support the formation of a cognitive map^13^^,^^23^ and construct an internal model of the subject’s “task space.”^24^ A task space is an abstract mapping of non-observable, causal relationships between the elements involved in a task (e.g., sensory cues, actions, outcomes, internal drives), which are relevant for achieving the subject’s goals.^22^^,^^23^ Task space representations may be acquired by model-based reinforcement learning, which enables the embedding of specific stimulus-outcome and action-outcome relationships in a task-relevant state space^25^^,^^26^ in addition to less specific, cached values assigned to cues and actions. Task space representations may subserve goal-directed action and prospective cognition, and may be coded jointly in other structures such as medial prefrontal cortex (mPFC). Whereas the mPFC may be specialized in coding action-outcome relationships and corresponding task rules,^27^^,^^28^^,^^29^ the OFC may engage more broadly in coding relationships between cues, spatial context, and outcomes.^4^^,^^21^^,^^22^^,^^30^^,^^31^ A major outstanding question is whether the OFC plays an active role in storing and consolidating task space information, either within its internal synaptic matrices or by driving changes in synaptic networks of connected areas (e.g., basolateral amygdala^32^). Alternatively, the OFC may import information acquired and stored in other areas to process it “on the fly,” thereby controlling how downstream target areas deploy associatively learned information to steer behavior.^13^^,^^21^

The consolidation of spatial memory causally depends on hippocampal sharp-wave ripple activity, harboring offline replay of task-related information.^33^^,^^34^^,^^35^^,^^36^^,^^37^ However, it is unknown whether neuronal OFC ensembles also display reactivation of firing patterns characteristic of prior task performance during offline states (sleep and quiet wakefulness). If the OFC replays task-related information during offline states, its relationship with hippocampal replay would become a topic of special interest. In addition to supporting spatial memory consolidation,^38^^,^^39^ area CA1 replay is coordinated with target areas such as the ventral striatum,^40^ mPFC,^29^^,^^41^ medial entorhinal cortex,^42^ sensory cortex,^43^ and basolateral amygdala.^44^ On the one hand, the OFC is anatomically more remote from hippocampal area CA1 than these other areas, as it receives no (or minor) direct input from CA1.^45^^,^^46^ On the other hand, the OFC codes spatial and contextual aspects of goal-directed behavior,^30^^,^^47^^,^^48^ which suggests some form of hippocampal-orbitofrontal coordination subserving spatial memory. These contrasting arguments led us to investigate whether the OFC and CA1 jointly replay information to facilitate model-based learning in the limbic-affective cortico-basal ganglia loop they participate in.^22^^,^^49^

We studied OFC reactivation using a behavioral task performed on the steering wheel maze, where freely moving rats select spatial goal sites based on changeable place-reward couplings.^50^ Comparing offline periods before and after task performance, we asked: (i) do OFC ensembles show post-task reactivation during rest periods? (ii) Does reactivation strength cohere with changes in place-reward configuration that occurred during task performance preceding sleep? (iii) Does post-task reactivation strength correlate with changes in behavioral performance observed in a subsequent session? and (iv) Does OFC replay information coherently with activity in area CA1?

## Results

### Task acquisition

**Figure 1:**
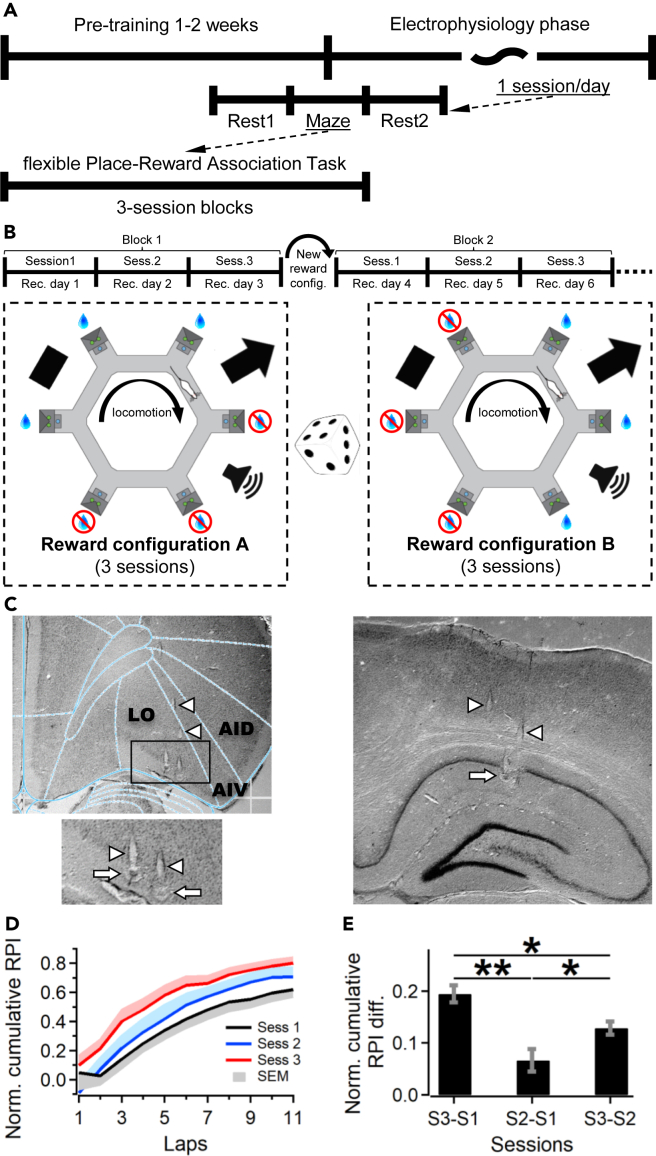
Task setup and behavioral training (A) Rats were pretrained on a hexagonal maze to obtain a sugar-water reward from six reward sites. Top: following a pre-electrophysiology training phase, where rewards were removed from three adjacent ports, animals were implanted with a tetrode microdrive. Middle: session structure. Bottom: during maze testing rats were subjected to a flexible place-reward association task. (B) Top: task structure using a block design, with each block spanning 3 sessions. Bottom: relative to distant geometrical cues (black rectangle, arrow, and loudspeaker) reward configurations (3 rewarded and 3 adjacent unrewarded ports) were kept unchanged for 3 consecutive sessions. After each block of 3 sessions, reward positions were changed pseudorandomly by 2–4 ports. (C) A split drive targeting the lateral OFC (LO, top) and the CA1 region of the hippocampus (right) was implanted after the pre-training phase. Bottom-left, zoom-in of the rectangular area outlined above. Arrowheads and arrows indicate tetrode tracks and tetrode tip locations, respectively; AID and AIV, dorsal and ventral parts of the agranular insular cortex. (D) Behavioral performance shown as a normalized, cumulative relative place-preference index (RPI) for each lap and preceding ones, calculated across the first 11 laps of each session. Data were averaged across all animals and session blocks. (E) Mean differences (±SEM) between normalized cumulative RPI across consecutive sessions in each block, calculated for all laps, ∗∗p < 0.01, ∗p < 0.05 - Bonferroni-corrected WMPSRT.

We measured the development of reward site preferences over the course of flexible place-reward association task (*fPRAT*, Figures 1A and 1B) sessions using a relative preference index (RPI). The RPI was calculated as the relative difference between the number of valid trials at rewarded and unrewarded ports in each lap around the maze, with valid trials represented by reward port visits where rats maintained a poke for a pre-set time interval (see STAR methods). We performed a block-based analysis where each training block spanned three consecutive sessions (1 session/day) in which reward positions were kept constant relative to distant geometrical cues. To measure acquisition performance within blocks, RPIs were averaged for each session across all blocks (Figure 1D). Across the three sessions of a block, rats displayed exponential place-preference curves with roughly similar asymptotic levels, but slightly different preferences at the start of the session (Figures 1D and S1). After the first two laps, we observed significant increases in session-to-session performance. These are reflected in differences between preference indices calculated for laps 1–11, with the highest and lowest preference values in session 3 (S3) and 1 (S1), respectively (difference S3-S1: 0.195 ± 0.017, S2-S1: 0.066 ± 0.021, S3-S2: 0.129 ± 0.012, S3-S1 vs. S2-S1 p < 0.01, S3-S1 vs. S3-S2 and S2-S1 vs. S3-S2 p < 0.05, Bonferroni-corrected Wilcoxon rank-sum test, WRST, Figure 1E). Thus, animals displayed progressive acquisition of place-reward associations across the three sessions of a block. Furthermore, differences between S3 and S1 calculated across the subset of relevant blocks did not increase as testing progressed, indicating that the task structure was reliably acquired during the pre-training phase.

### Orbitofrontal reactivation during rest episodes

**Figure 2:**
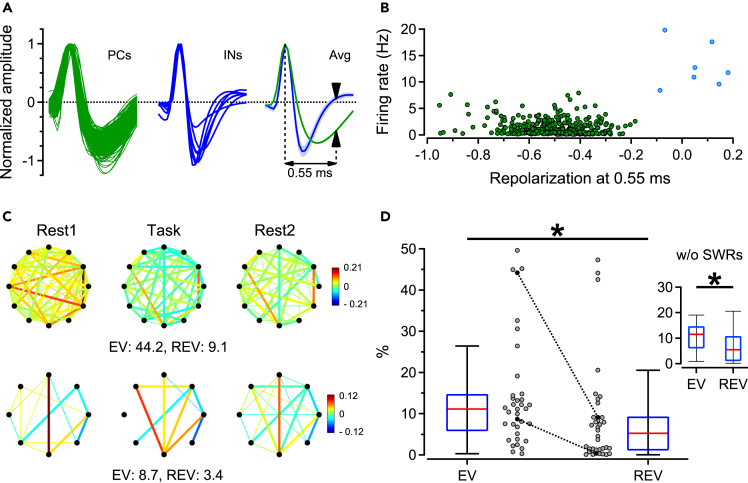
Reactivation in orbitofrontal cortex during rest (A) Normalized spike waveforms of putative pyramidal cells (PCs, green, left) and interneurons (INs, blue, middle) recorded from the OFC, and their corresponding averages (Avg, right). Arrowheads indicate waveform amplitude at 0.55 ms from peak. (B) Classification of OFC single units into putative pyramidal cells (green) and interneurons (blue), based on firing rate and repolarization amplitude. (C) Patterns of correlations between cell pairs measured during *Rest1*, *Task*, and *Rest2* for two example sessions (2^nd^ session of the 2^nd^ block and the 1^st^ session of the 6^th^ block); the 2^nd^ session was part of a block with 12 units (top), and the 1^st^ session was in a block with 8 units (bottom). The color map shows correlation strength and sign, and thick lines indicate cell pairs which were correlated during Task and also during Rest1 or Rest2. Upper panel: note how negative correlations during *Rest2* contribute to pattern similarity relative to Task. (D) Variance of correlation patterns observed during post-task rest, explained by correlations found during Task and controlled for pre-existing correlations as found during *Rest1* (explained variance: EV), versus the reverse explained variance (REV). Gray circles and boxplots show values for individual sessions and Q1, median and Q3, respectively; connected black circles are example sessions shown in C. Inset shows EV and REV values calculated using the same dataset, except that spikes recorded during SWRs were eliminated. WMPSRT: ∗p < 0.05.

To examine electrophysiological mechanisms supporting consolidation of place-reward associations in the OFC, we asked whether correlation patterns newly established between single units during the task are reinstated during subsequent offline states. Spike correlations for all pairs of OFC putative pyramidal neurons, as determined by their slower repolarization and lower firing rate relative to putative fast-spiking interneurons (Figures 2A and 2B), were calculated across pre-task rest (*Rest1*), task, and post-task rest (*Rest2*) episodes (Figure 2C; for pooled OFC-CA1 data, see Figure S2). Reactivation was estimated using explained variance (EV) analysis,^33^ taking the temporally mirrored measure, the reversed explained variance (REV), as a control.^51^ As demonstrated by a larger median EV (11.1) relative to the REV (5.3, Wilcoxon matched-pairs signed-rank test - WMPSRT: p = 0.016, 36 sessions from 3 rats, Figure 2D), functional relationships expressed during *Task*, but not during *Rest1*, were reactivated during *Rest2*. This difference was maintained when spikes recorded during hippocampal sharp-wave ripples (SWRs) were eliminated from the dataset (EV = 11.5 vs. REV = 5.5, WMPSRT: p = 0.021). These results indicate that reactivation of task-related firing patterns is present in OFC, even in the absence of ripples.

### Spatiotemporal correlates of memory trace reactivation in orbitofrontal cortex

**Figure 3:**
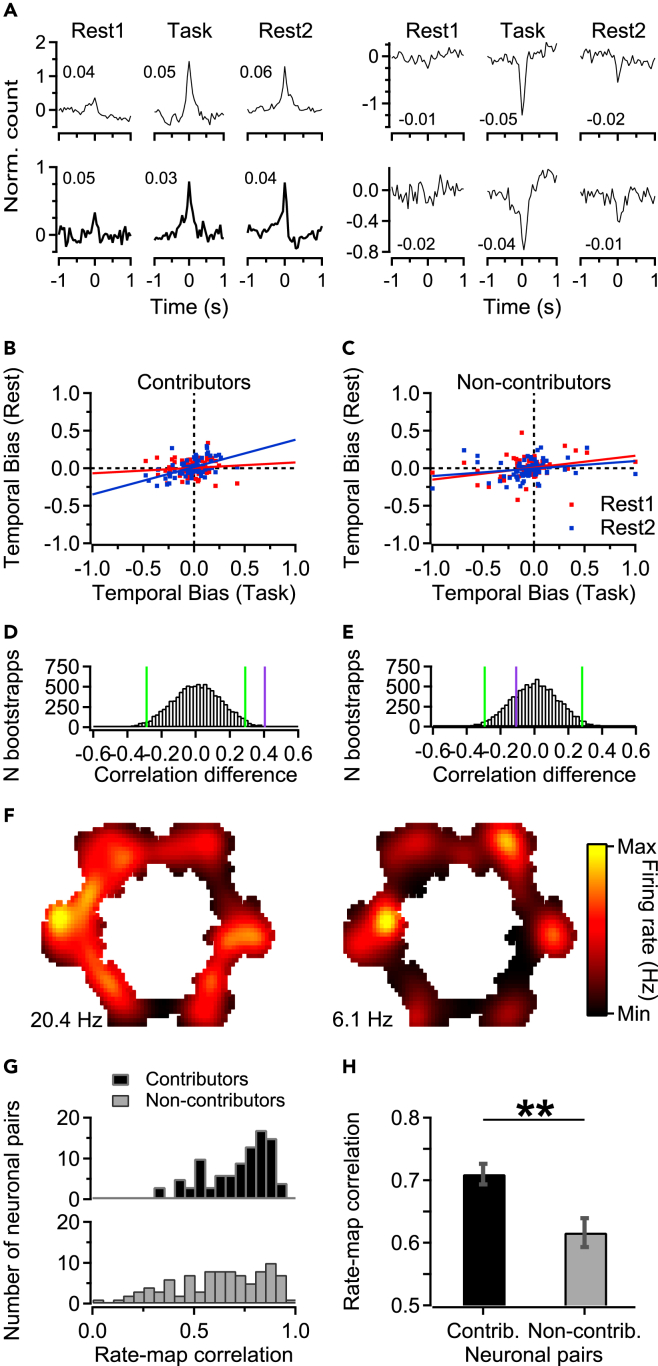
Orbitofrontal cell pairs contributing to reactivation show different temporal biases and rate-map correlations than non-contributing pairs (A) Cross-correlograms of normalized spike counts versus time lag for four example OFC cell pairs during *Rest1*, *Task*, and *Rest2,* showing either positive (left) or negative (right) spike time relationships. Insets show linear correlation coefficients (Pearson’s r across continuous spike trains, quantified in 50 ms bins) between spike patterns of the corresponding example cell pair, for each episode. (B) Significant difference in linear correlations between temporal bias scores for *Rest2*-*Task* (blue) and *Rest1*-*Task* (red) in pairs strongly contributing to reactivation (estimated using jackknifing, bootstrap statistics: p < 0.01). (C) This difference is absent in non-contributors. (D and E) Histograms of bootstrapped correlation differences (between *Rest2*-*Task* versus *Rest1*-*Task*) and significance levels for 2.5% and 97.5% (green lines), corresponding to contributors (D) and non-contributors (E). Purple lines indicate differences between correlation coefficients measured for the non-bootstrapped data in B and C. In D but not E, the purple line indicates a significantly stronger difference between correlation coefficients than expected from bootstrapping (p < 0.05). (F) Rate maps for a pair of putative pyramidal neurons in OFC contributing to reactivation (rate map correlation: 0.74). Numbers indicate maximum firing rates (lower left). (G) Distributions of correlations between rate maps of neuronal pairs classified as contributors (black, top) and non-contributors (gray, bottom). Note the right-shifted distribution of contributor correlations. (H) Mean (±SEM) calculated for Pearson’s correlation coefficients shown in G, WRST: ∗∗p < 0.01.

Previous studies have shown that task variables (e.g., rewards, cues, locations) and their spatiotemporal succession constrain neural activity patterns during post-task consolidation and replay. For instance, hippocampal, ventral striatal, and visual cortex ensembles fire in specific sequences which are largely conserved during rest periods in between sessions.^40^^,^^43^^,^^52^^,^^53^ Similarly, *fPRAT* has a sequential structure where clockwise visits can be reinforced at six spatially distinct ports with variable reward contingencies. The clockwise running direction enabled us to investigate whether temporal associations between neuronal pairs, expressed during the task, are reactivated during post-task rest. Thus, we computed a temporal bias (T_B_) score (Figure 3A), based on cross-correlograms for all cell pairs recorded in each session.^54^ T_B_ scores, calculated as the relative differences between cross-correlogram integrals in a 400 ms time window on either side of t = 0 s, were pooled across all sessions. Consistent with hippocampal and striatal recordings,^40^^,^^54^^,^^55^^,^^56^^,^^57^ a Pearson’s correlation analysis of T_B_ scores computed for *Rest2* vs. *Task* yielded higher mean correlations than T_B_ scores for *Rest1* vs. *Task* (*Rest1* vs. *Task*, r_Rest1_ (d.f.: 2581) = 0.088, p < 0.001; *Rest2* vs. *Task*: r_Rest2_ (d.f.: 2581) = 0.143, p < 0.001). A bootstrap analysis that resampled T_B_ scores across task episodes revealed that these T_B_ correlation differences were statistically significant (p = 0.02). Thus, the temporal structure of pairwise relationships in OFC during task performance is significantly reinstated during post-task as compared to pre-task rest.

A reinstatement of OFC temporal structure during post-task rest may be identifiable only in a subset of the task-engaged ensembles^58^ or in “engram neurons.”^59^^,^^60^ To investigate whether the sequential encoding of task events shapes neuronal activity during post-task rest generally or by way of subsets, we restricted analyses to sessions with a positive reactivation strength (i.e., (EV-REV) > 0) and constructed a distribution of cell-pair contributions to reactivation using jackknifing.^56^ These contributions were quantified by estimating EV and REV with and without the pair under scrutiny and taking the difference in (EV-REV) as the pair’s contribution to reactivation over an entire session. Next, we compared T_B_ correlations for positive and strong reactivation contributors versus non-contributors, i.e., the cell pairs within the highest and lowest 5^th^ percentile of the contribution distribution. Whereas a strong (EV-REV) contribution by itself does not reveal a temporal ordering of spike patterns of pair members, strong contributors are predicted to show such ordering by way of temporal asymmetries in their cross-correlograms. Contributor pairs displayed a significantly higher T_B_ correlation between *Rest2* and *Task* than *Rest1* and *Task* (r_Rest2_ (d.f.: 91) = 0.51 vs. r_Rest1_ (d.f.: 91) = 0.11, Figure 3B; Bootstrap test: p < 0.01, Figure 3D). In contrast, neuronal pairs with low contributions showed similar relationships between T_B_ scores of the two rest phases and *Task* (r_Rest2_(d.f.: 91) = 0.20 vs. r_Rest1_(d.f.: 91) = 0.31, Figure 3C; Bootstrap test: p = 0.78, Figure 3E). Similar differences were observed for the top and bottom 2.5 percentiles (non-contributors: r_Rest2_(d.f.:45) = 0.001 vs. r_Rest1_(d.f.:45) = 0.28, Bootstrap test: p = 0.91; contributors: r_Rest2_(d.f.:45) = 0.46 vs. r_Rest1_(d.f.:45) = −0.16, Bootstrap test: p < 0.01) and 7.5 percentiles (non-contributors: r_Rest2_(d.f.:138) = 0.27 vs. r_Rest1_(d.f.:138) = 0.13, Bootstrap test: p = 0.12; contributors: r_Rest2_(d.f.:138) = 0.40 vs. r_Rest1_(d.f.:138) = 0.14, Bootstrap test: p < 0.05) of the contribution distribution. These results suggest that information about the temporal task sequence is preferentially replayed by some neuronal subsets in OFC more than by others, underwriting the heterogeneous nature of OFC populations in task-related replay.

Not only the temporal order of *fPRAT* events but also representations of place-reward configurations on the steering wheel maze may be actively re-expressed during offline replay. To investigate this, we computed firing-rate maps for all putative OFC pyramidal neurons (see examples in Figure 3F). Given results from previous studies,^30^^,^^61^ it was surprising to find that the spatial selectivity and spatial information content of OFC cells (n = 424) were low (3.70 ± 0.13 and 0.29 ± 0.01 bits/spike, respectively), when compared to CA1 cells recorded in the same experiment (6.04 ± 1.13 and 1.76 ± 0.09 bits/spike, p < 0.001, n = 74). Nonetheless, when we plotted distributions of rate-map correlations for contributors vs. non-contributor pairs, we observed a rightward shift in the distribution of contributors relative to non-contributors (Figure 3G). Thus, OFC cell pairs that strongly contributed to reactivation showed higher rate-map overlap than non-contributors (mean rate map correlation - contributors: 0.71 vs. non-contributors: 0.62, WRST: p < 0.01, Figure 3H).

These findings were not paralleled by a difference in overall spiking activity between contributor and non-contributors, as shown by similar firing rates measured during offline and task periods (contributors vs. non-contributors: Rest1: 1.65 ± 0.16 Hz vs. 1.47 ± 0.17 Hz, p = 0.41 (d.f. 175); Task: 1.98 ± 0.20 vs. 1.64 ± 1.18, p = 0.24, d.f. 175; Rest2: 1.63 ± 1.16 vs. 1.36 ± 0.15, p = 0.21, d.f. 175; Bonferroni-corrected WMPSRT). In addition, the quality of spike clusters representing contributor and non-contributor units did not show significant differences when isolation distance and L-ratio^62^ were compared between the two categories (contributors vs. non-contributors: L-ratio: 0.061 ± 0.011 vs. 0.076 ± 0.010, p = 0.30; isolation distance: 35.8 ± 2.4 vs. 30.9 ± 1.7, p = 0.14, WRST). Moreover, the two groups of units did not show a difference in burstiness (Figure S4).

Taken together, these differences in spatiotemporal relationships between replay contributor and non-contributor OFC cell pairs indicate that during post-task rest, a subset of OFC neurons replays spatiotemporal patterns characteristic of the sequential nature of the task.

### Strength of orbitofrontal reactivation depends on the recency of changes in place-reward configuration

**Figure 4:**
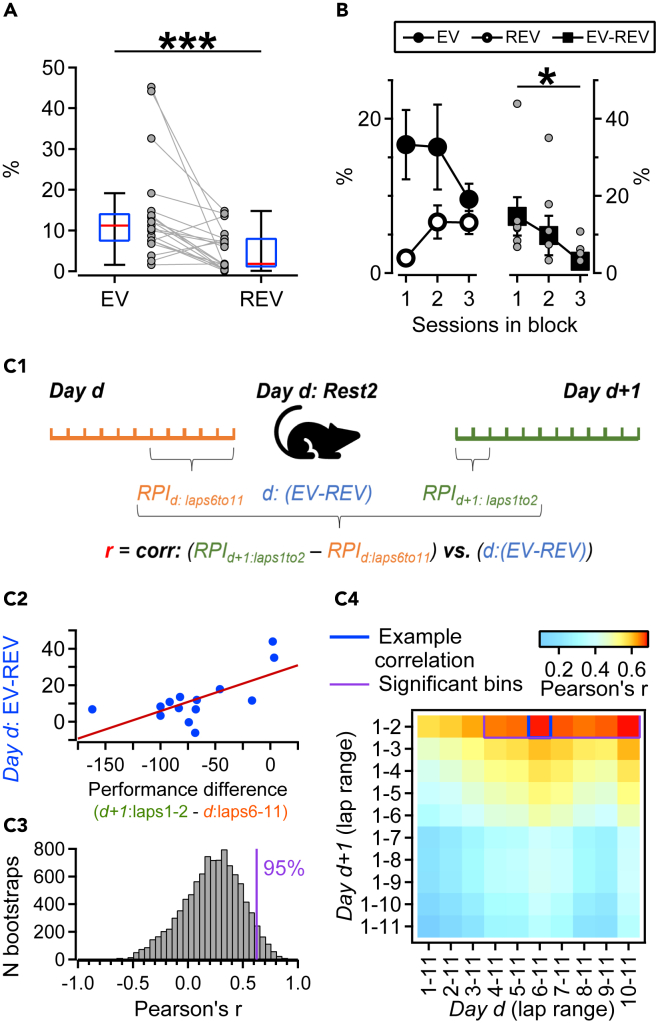
Orbitofrontal reactivation depends on recency of changes in place-reward configuration and task performance (A) Boxplots (Q1, median, Q3) show explained and reversed explained variance (EV and REV) calculated for 21 sessions corresponding to 7 complete blocks using OFC units. Gray markers and lines show data from individual sessions, WMPSRT: ∗∗∗p < 0.001. (B) Left: Mean EV and REV calculated by session rank across blocks; Right: corresponding mean (black squares) and single-session differences (gray circles) between EV and REV (reactivation strength). Reactivation strength decreased significantly as a function of change recency (Friedman’s test: p < 0.05, Bonferroni-corrected WMPSRT: p < 0.05). (C1) Schematic illustrating the calculation of the Pearson’s correlation coefficient shown in C2 and C4 (blue square). Correlations between the reactivation strength on a given session (*Day d*) and differences in behavioral performance between the subsequent session (*Day d+1*) and the current one for which reactivation strength was computed, calculated for lap ranges 1–2 and 6–11, on *Day d+1* (all sessions 2 and 3) and *Day d* (all sessions 1 and 2), respectively. All 14 *Day d* and *d+1* pairs were used. RPI: relative place preference index. (C2) Correlation between performance (RPI) differences and reactivation strengths (r = 0.67, p = 0.008). The performance difference between the initial laps on *Day d+1* and laps 6–11 on *Day d* was negative in a majority of cases, presumably because the initial laps on *Day d+1* may involve more exploratory behavior and delay in reinstating task set as compared to performance in later trials. The higher the reactivation strength on *Day d*, the easier it was for rats to quickly achieve similar performance on *Day d+*1 relative to *Day d*. (C3) Distribution of correlations between reactivation strength and performance change computed by bootstrapping lap identity (for all *Day d* and *d+1* pairs and all lap ranges). The purple line indicates Pearson’s correlation coefficient at the 95^th^ percentile (r = 0.63). (C4) Correlations between reactivation strength and performance difference, calculated for different lap ranges on *Day d* (abscissa) and *Day d+1* (ordinate). Blue and purple contours indicate the example in C2, and significant (p < 0.05) Pearson’s r values estimated using the bootstrap test in C3, respectively. This graph raises empirical evidence for a significant relationship between reactivation after the task performed on *Day d* and the initial behavioral performance on the next day *d+1*, corrected for the RPI on *Day d*.

We next asked whether OFC reactivation strength depends on the repeated exposure of the rat to a particular place-reward configuration on the maze, and may thus be related to the updating of place-reward associations. Therefore, we examined reactivation separately for the three sessions making up a training block. To compare sessions sharing the same reward configuration, this analysis considered only blocks containing all three consecutive sessions (7 blocks containing 21 sessions). In this data subset, we again found reactivation of OFC task patterns during post-task rest: the EV exceeded the REV (median of all 21 sessions: EV = 11.2 vs. REV = 1.8, WMPSRT: p < 0.001, Figure 4A). Across the 3-session blocks, the largest EV and smallest REV, and thus the maximum reactivation strength, were found in the first session (Figure 4B left). A decrease was observed within blocks (mean differences: EV-REV_S1_ = 14.7 ± 5.0, EV-REV_S2_ = 9.7 ± 5.1, EV-REV_S3_ = 3.0 ± 2.1, Friedman’s test: p = 0.028, Figure 4B right) with a significant difference in reactivation strength between the first and third session (p = 0.047, Bonferroni-corrected WMPSRT). Thus, the relative novelty of place-reward coupling in a familiar environment favors strong reactivation in the OFC network.

### Orbitofrontal reactivation strength coheres with overnight change in behavioral performance

So far, these results indicate that task-related information, recently acquired during maze experience, is reactivated by OFC cell ensembles. The extent to which replay coheres with performance may be detectable in the correlations between the overnight changes in behavioral performance across consecutive maze runs and neural activity during interleaved rest episodes. To test this, we calculated the overnight performance difference (i.e., the difference in place preference index between pairs of consecutive sessions, for different temporal segments of the task period), and correlated this difference with the reactivation strength observed after the first fPRAT session of this session pair (see STAR methods, Figure 4C1). Thus, we obtained a 10 x 10 reactivation-behavioral correlation (RBC) matrix in which each element is the Pearson’s correlation coefficient *(r)* for the reactivation strength (i.e., EV-REV) on *Day d* versus the RPI difference between *Day d+1* and *Day d*, for a given subset of laps (Figure 4C). Overall, differences between RPI during the initial laps on *Day d+1* and the later series of laps on *Day d* showed significant linear correlations with replay strength (see example in Figure 4C2, and 4C4). A bootstrap test with randomized lap assignment showed that a subset of correlation matrix elements, corresponding to laps 1–2 on *Day d+1* and laps 4–11 on *Day d* (Figures 4C3, and 4C4; purple rectangle), fell in the upper 5^th^ percentile of the bootstrapped distribution (Figure 4C3, *r*_95%_ = 0.63). In addition, we tested other parameter combinations, such as EV vs. the difference in performance described in Figure 4 and, to compare equal numbers of laps on consecutive days, we built similar RBC matrices by correlating EV or the EV-REV difference with the difference in performance between moving intervals of three laps on day D+1 - day D (data not shown). All analyses showed a correlation profile similar to Figure 4C4. This grouping indicates that the strength of OFC reactivation correlates with the performance difference between consecutive sessions, supporting the notion that OFC replay in between subsequent learning episodes contributes to changes in task performance.

### State-dependent coordination of orbitofrontal replay with hippocampus

During spatial tasks, neural activity in the OFC is synchronized to CA1 activity, particularly in the theta band following learning and at decision points.^48^ Together with recent evidence showing that optogenetic inhibition of the subiculum alters single-cell coding of response direction and OFC representations of outcome-relevant blocks of trials,^63^ these findings suggest that hippocampal-prefrontal interactions may be critically involved in adaptation to changing reward associations. Despite the presence of OFC replay in the absence of hippocampal SWRs, we thus asked whether dorsal area CA1 and OFC jointly reactivate after *fPRAT* performance.

**Figure 5:**
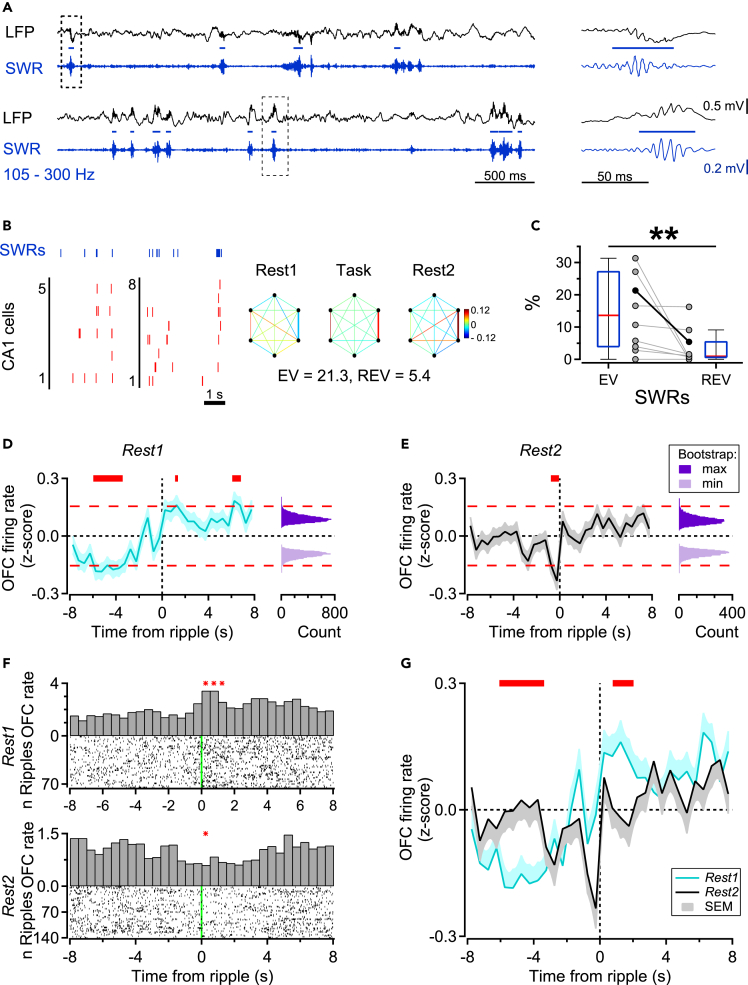
Modulation of orbitofrontal firing activity is temporally coordinated with hippocampal ripples during rest (A) Left, example CA1 LFP (black) and SWR (blue) traces from two sessions, blue horizontal bars indicate SWRs, as detected in the bottom blue trace. Dashed rectangles indicate time segments enlarged in the panels on the right. (B) Left, example cell sequences recorded during QW-NREM (red bars: CA1 spikes, blue: SWRs) from two recording sessions (left and right). Examples, left and right, are taken from the same recordings as shown in A, top and bottom, respectively. Right, patterns of correlations between CA1 cell pairs measured during *Rest1*, *Task*, and *Rest2* for an example session with six simultaneously recorded hippocampal neurons. The color map shows correlation strength and thick lines indicate cell pairs which were correlated during Task and also during Rest1 or Rest2. (C) Reactivation of firing patterns in CA1 during SWRs, indicated by EV and REV values across sessions (boxplots: Q1, median, Q3) and single session data (gray markers and lines, black shows the session in B, n = 10 sessions, WMPSRT, ∗∗p < 0.01). (D and E) Averages of firing rates during *Rest1* and *Rest2*, respectively, for all neurons recorded in OFC, aligned on hippocampal SWR onset. Interrupted red lines indicate upper and lower significance levels. Thick horizontal red lines indicate statistically significant differences between PRTH time points and top and bottom 5% of maximum and minimum bootstrapped distributions, respectively. (F) Top and bottom, examples of peri-ripple time histograms (PRTHs) for two OFC cells during Rest1 and Rest2, respectively; red asterisks indicate significant bins, ∗p < 0.05, Wilcoxon’s matched-pairs signed-rank tests. (G) *Z* scored average PRTHs shown in D and E. Thick horizontal red lines indicate statistically significant differences between PRTHs computed across *Rest1* (cyan) and *Rest2* (black) episodes, determined using a cluster-based permutation test (p < 0.05).

As expected, ensemble activity in area CA1 was highly synchronized during SWR complexes (Figure 5B left). Reactivation in dorsal area CA1 prominently occurs during SWRs recorded in quiet wakefulness and non-REM sleep (QW-NREM) episodes,^33^ and this was also the case following *fPRAT* (CA1: EV = 13.6 vs. REV = 0.9, WMPSRT: p < 0.01; n = 10 sessions, Figure 5B right, C). Even though the OFC is anatomically remote from CA1, we found that OFC firing activity was entrained to hippocampal SWRs on a timescale of seconds, both during pre- and post-task rest (Figures 5D and 5E; cf;).^57^ Peri-ripple time histograms (PRTHs) showed a significant decrease in mean OFC firing rate for several seconds preceding SWRs, followed by a gradual rise, peaking in a 1 s time-window following ripple onset during pre-task rest (bootstrap: p < 0.01 Figure 5D; for example OFC units, see Figure 5F; WMPSRT: p < 0.05). During post-task rest, OFC firing generally displayed a pattern similar to pre-task rest, but now OFC unit activity was downregulated more sharply before ripple onset (Figure 5E). A comparison between *Rest1* and *Rest2* PRTHs of OFC neurons showed a more pronounced trough and peak around SWRs in *Rest1* relative to *Rest2* (Figure 5G, cluster permutation test: p < 0.05). However, when correlations between CA1 and OFC cells were compared for all SWRs during *Rest1* and *Rest2*, no overall reactivation across the OFC-CA1 network could be detected (EV = 1.3 ± 0.5 vs. REV = 1.8 ± 0.8, WMPSRT: p = 0.105).

**Figure 6:**
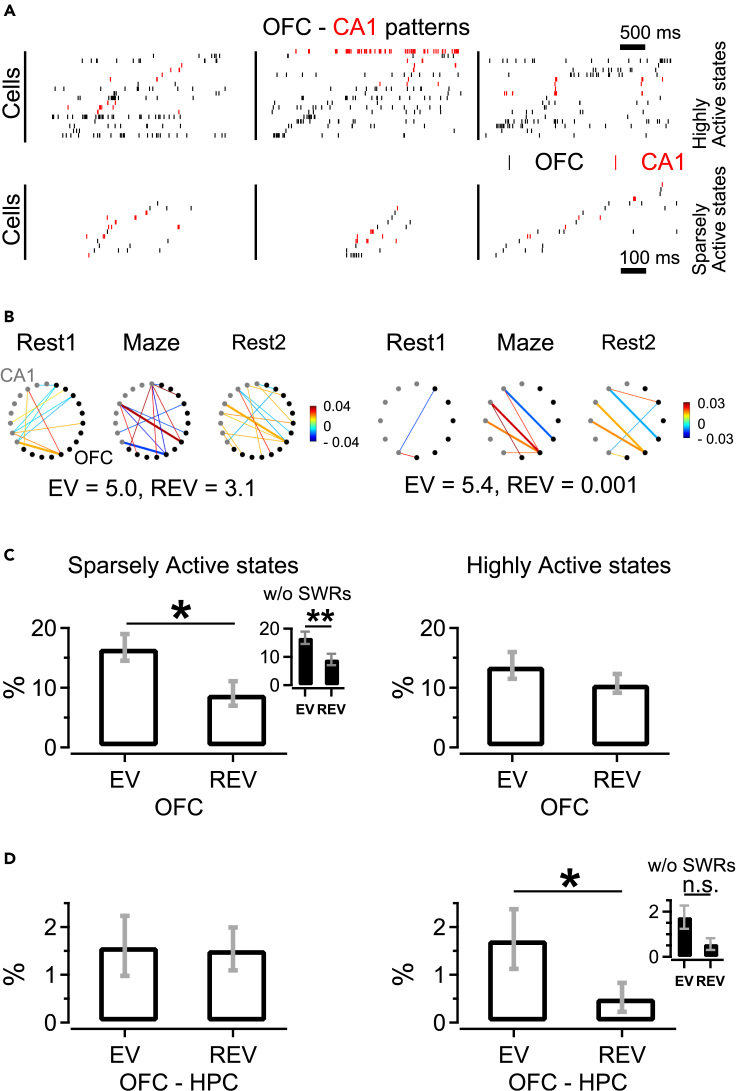
Orbitofrontal reactivation and joint orbitofrontal-hippocampal reactivation occur during different brain states (A) OFC-CA1 cell sequences recorded during three sessions (columns). For each example sequence, Highly (top) and Sparsely Active states (bottom) are shown for the same spiking cells (red - CA1 units, black - OFC units). Each panel shows spikes from all active cells in each example Active state, sorted by peak firing rate time within each respective state. (B) Patterns of correlations between OFC (black circles) and CA1 (gray circles) cell pairs computed for Highly Active states across all task episodes from two example sessions. The color bar shows correlation strength and thick lines indicate cell pairs correlated during Task and also during Rest1 or Rest2. (C) EV and REV calculated during Sparsely and Highly Active states (left and right, respectively) identified in *Rest1* and *Rest2* intervals, for OFC neuronal ensembles (36 sessions). (D) Same as C, except that the variance was computed for OFC-CA1 ensembles (10 sessions). Inset shows EV and REV computed for Highly Active states without spikes fired during hippocampal SWRs; ∗p < 0.05, Bonferroni-corrected WMPSRT, n.s. not significant.

We next fractionated OFC activity in relation to reactivation. This fractionation was inspired by the distinction between Up and Down states,^64^ but was not based on the presence of slow-wave activity preceding or following a period of enhanced firing activity, as would be the case for Up states. Thus, we distinguished Active states by a more inclusive measure based on the graded distribution of spike counts as compared to the definition of (slow-wave activity coupled) Up states. After identifying Active state segments from OFC multiunit activity (based on spike counts during intervals of sustained activity; see STAR methods), these states were subdivided into quartiles, where we focused on the first and last quartiles. The first quartile of Active states is labeled “Sparsely Active state” here, with the number of spikes thus being in the lowest 25^th^ percentile of the Active state distribution computed for the number of spikes per state. The last quartile of Active states is called “Highly Active state”, representing the highest 25^th^ percentile of this distribution (Figure S3A, left). Figure 6A shows examples of synchronized unit firing in both state types across OFC and CA1. Next, we estimated intra- and inter-area reactivation across these two different types of Active state for OFC and OFC-CA1 pairs of neurons (see Figure 6B for two examples of Highly Active state). Although Active states showed a substantial overlap (61%) with Up states as defined by Vyazovskiy et al.,^64^ we note that, in our data, Up states formed a subset of Active states (see STAR methods), which are the states referred to in the current work.

Remarkably, intra-OFC replay preferentially occurred during Sparsely Active states during post-task rest (EV = 16.7 ± 2.3% vs. REV = 9.0 ± 2.1%, Bonferroni-corrected WMPSRT: p < 0.05, Figure 6C left). OFC reactivation was still observed when spikes fired during CA1 SWRs were eliminated from the dataset (EV = 16.7% ± 2.2% vs. REV = 9.1% ± 2.0%, WMPSRT: p < 0.01). In contrast, the (EV-REV) difference for OFC was not significant for Highly Active states (EV = 13.7% ± 2.2% vs. REV = 10.7% ± 1.6%, WMPSRT: Figure 6C right). An opposite relationship, however, was observed for inter-area replay. While the EV-REV difference for OFC-CA1 pairs during Sparsely Active states was not significant (EV = 1.6 ± 0.6 vs. REV = 1.5 ± 0.5, WMPSRT, Figure 6D left), task-related patterns were reinstated in OFC-CA1 pairs during Highly Active states (EV = 1.8 ± 0.6 vs. REV = 0.5 ± 0.3, WMPSRT: p < 0.05, Figure 6D right, B). Similar patterns in reactivation strength were observed when EV and REV were calculated using OFC and OFC-CA1 unit pairs for different distribution percentiles of Active states (Figure S3). A trend toward joint OFC-CA1 reactivation was observed when SWR spikes were eliminated from both OFC and CA1 spike vectors, but this was not significant (EV = 1.8 ± 0.5 vs. REV = 0.6 ± 0.3, WMPSRT: n.s., Figure 6D). In general, inter-area EV and REV values were low. The sparse and predominantly polysynaptic connections between CA1 and OFC may translate functionally in low neuronal activity correlations across these regions.^65^^,^^66^ Indeed, the mean Pearson’s correlation coefficient of OFC-CA1 pairs in the Highly Active state was in the range of one order of magnitude smaller than that for OFC pairs during QW-NREM, or CA1 pairs during SWRs (OFC-CA1: 0.0048 ± 0.0003 vs. OFC: 0.0168 ± 0.0005, WRST: p < 0.001; or vs. CA1: 0.0377 ± 0.0045, WRST: p < 0.001). These data suggest that OFC ensembles engage in different replay relationships during distinct brain states: during Sparsely Active states they engage primarily in local, intra-areal replay, whereas during Highly Active states they reactivate together with CA1 ensembles.

## Discussion

OFC ensembles were shown to reactivate task-related information patterns during offline states following search behavior on a steering wheel maze involving variable place-reward contingencies. Neuronal pairs strongly contributing to replay displayed similar temporal and spatial relationships during post-task rest as during active behavior. Notably, reactivation was stronger after task sessions in which place-reward couplings were changed relative to the preceding session block, and reactivation strength correlated with a measure of the overnight change in behavioral performance. OFC firing patterns were differentially modulated by SWR activity during pre- and post-task rest. Whereas ensembles within OFC showed significant reactivation during Sparsely Active states, joint OFC-hippocampal reactivation was selectively found during Highly Active states.

### Orbitofrontal reactivation and the updating of place-reward associations

We found that OFC reactivation strength depended on the recency of a shift in place-reward coupling on the maze. When recording rats that had been exposed to a shift in place-reward configuration, OFC post-task reactivation was stronger in the first session than in the two subsequent sessions of the same block having the same configuration (Figure 4B). On average, a decrease in performance was observed when comparing the final laps on *Day D* to the initial laps on *Day D+1*, which may be attributed to adopting a more exploratory strategy at the start of a new session and a concomitant delay in the reinstatement of task set.^3^^,^^67^^,^^68^ In addition, the levels of arousal, motivation, and attention may change over the course of a session. Nevertheless, when correlating the RPI change from *Day d* to *Day d+1* of the same block to the reactivation strength on *Day d*, we found a strong positive correlation (i.e., when the RPI on *Day d+1* was restricted to the first few laps). Thus, a strong reactivation on *Day d* was especially associated with a lack of performance drop at the start of the session on *Day d+1*, and can be interpreted as a successful reinstatement of learned fPRAT associations as recalled from the previous session (Figure 4C). That the correlations declined when more laps of *Day d+1* were included can be explained by a multitude of factors that may affect behavioral performance on this day, and may well be causally and historically unrelated to the preceding day (e.g., different levels of arousal, motivation, and attention as compared to the previous day, new learning, or intrusion of remote memories).

What we consider especially striking in the boosted OFC reactivation after a shift in place-reward coupling is that the level of task context novelty and required behavioral adaptation in this situation was relatively limited. Hippocampal reactivation was previously reported to be enhanced in a novel environment.^55^ In contrast, the OFC reactivation we report was enhanced within a familiar environment. No novel reward sites were introduced in our task; each of the six goal sites on the maze had been rewarded at some point in the animal’s training history. We also did not introduce a novel task rule, but required animals to update previously learned place-reward associations as these changed from block to block. The importance of this distinction is underwritten by previous findings showing that mPFC lesions in marmosets impair extradimensional shifting but not reversal learning, whereas OFC lesions produce the opposite pattern.^3^ The place-reward updating studied here largely conforms to the principle of reversal learning, because upon a shift, a subset of previously rewarded places was no longer rewarded, whereas a subset of non-rewarded places became coupled to reward. The relationship between place-reward updating (Figures 1D and 4) and OFC reactivation (Figure 4) is thus consistent with its causal function in reversal learning^5^; but see below,^22^.

### Properties of orbitofrontal reactivation in relation to memory consolidation

Cell pairs that strongly contributed to reactivation showed a preservation of temporal bias relationships from *Task* to *Rest2* relative to *Rest1*. This result underscores that reactivation is not only manifested by way of binned cross-correlations (Figure 2) but is also characterized as a (temporally specific) replay. Moreover, spatial correlations between cell pairs were significantly higher for contributor pairs than non-contributors (Figures 3F–3H). Intrinsic firing properties of contributor cells are unlikely to underlie these correlations. Contributor cells did not show an increase in firing rate during Rest2, and if they would have shown a more spatially or temporally confined firing irrespective of task activity, high firing-rate correlations would be observed during Rest1 as well, which would be reflected in a decreased reactivation contribution score. Both the temporal and spatial correlation patterns confirm that replay depends on the specific relationships between OFC cells as they “tessellate” the unidirectional sequence of running-and-foraging during each session.^69^^,^^70^ Superficially, these OFC results resemble reactivation characteristics reported for the hippocampus,^54^ ventral striatum,^40^ neocortex,^41^^,^^42^ and other structures.

In comparison to area CA1 and the hippocampal-ventral striatal network, however, most OFC cell pairs showed synchronous and temporally symmetric activation patterns, resulting in a distribution of relatively low temporal biases centered around zero (Figures 3A–3E). Furthermore, cross-correlograms during Rest2 did not reveal a clear temporal compression of pairwise firing relationships, unlike the compressed, ripple-associated replay reported for hippocampus,^52^^,^^53^^,^^71^ mPFC,^29^^,^^41^ and ventral striatum.^40^ Rather, OFC patterns resemble the uncompressed offline reactivation as reported for putative dopamine neurons in the ventral tegmental area (VTA).^72^^,^^73^ Importantly, OFC reactivation persisted when removing OFC spikes recorded during hippocampal ripples (Figure 2D), whereas reactivation in ventral striatum and mPFC coheres with SWR activity.^29^^,^^40^ These combined findings suggest that the OFC and VTA may be part of a motivational network sustaining offline reactivation independently of the hippocampus, at least in particular brain states.

Memory formation and consolidation are thought to be supported by Hebbian mechanisms regulating synaptic plasticity, often involving NMDA receptors.^74^^,^^75^^,^^76^ In the OFC, local NMDA receptors have been causally implicated in synaptic plasticity underlying the formation of ensembles discriminating sensory cues paired with different outcome values.^77^ We hypothesize that this mechanism may extend to offline consolidation phases such as post-task sleep and rest. Indeed, studies on hippocampus and mPFC have implicated NMDA receptors in post-task memory reactivation and retention.^78^^,^^79^ In hippocampus-dependent consolidation, SWRs may function to accelerate replay and boost NMDA receptor-dependent plasticity.^51^^,^^78^ Because in our study OFC reactivation occurred independently of SWRs, a boosting effect based on temporal compression may be absent in OFC. Conceivably, the hippocampus plays a special role in memory consolidation as a “fast absorber” of information vis-à-vis neocortical structures, which would incorporate new information more slowly to be protected against catastrophic interference.^80^^,^^81^ Mechanistically, the OFC offers the opportunity to study how SWR-independent replay processes cohere with the speed and efficacy of memory consolidation, and whether the involved transmitter and receptor systems are different from those implied in SWR-dependent consolidation. In addition to NMDA receptors, especially dopamine and serotonin (5-HT2C) receptors will be interesting to study in the context of cognitive OFC functions.^82^^,^^83^^,^^84^

### Co-reactivation of orbitofrontal and hippocampal ensembles

Despite the finding that OFC reactivation occurred in the absence of hippocampal SWR activity, two observations suggest that OFC firing activity is conditionally coordinated with CA1 activity: (i) OFC firing activity was modulated in association with SWRs on a timescale of seconds, and this modulation differed between pre- and post-task rest (Figure 5G), and (ii) joint OFC-hippocampal replay did occur but was selectively found during Highly Active states (Figure 6D). This juxtaposition of findings may seem paradoxical given that SWRs and Down-to-Up state transitions in neocortex were previously reported to be correlated.^85^ However, this paradox may be explained by OFC-hippocampal coactivity being coordinated by other mechanisms than those directly depending on SWRs, while it is also relevant to recall that the Active states as defined here are not identical to Up states.^64^ Instead of being SWR dependent, OFC-hippocampal replay may be enabled by the very occurrence of Highly Active states, providing a temporal window for replay to emerge and unfold, whereas only their initial temporal segment may tightly cohere with SWR activity.^85^ As yet it is difficult to underpin why OFC-hippocampal replay occurred selectively during Highly Active states whereas intra-OFC reactivation was restricted to Sparsely Active states. These Sparsely Active states described here for OFC replay may be functionally similar to short duration, fast decorrelating Up-states reactivating recent memory traces in mPFC.^86^

Highly Active states may allow more time for extended network interactions to be recruited during non-REM sleep as compared to Sparsely Active states, which may only permit short-range and local network activations. This idea is compatible with findings indicating a general decline in long-range functional connectivity during non-REM sleep and anesthesia,^87^^,^^88^^,^^89^ as opposed to preservation of short-range connectivity.^90^ However, our results lead to the amendment that QW-NREM states may permit long-range interactions selectively during periods of high cortical excitability (such as Highly Active states), which may propagate in a fronto-temporal direction and entrain parahippocampal areas,^22^ while local reactivation predominates during shorter, Sparsely Active states.^86^

The conditional occurrence of joint OFC-hippocampal replay also raises the question through which anatomical pathways cross-structural coordination may occur. Area CA1 is not known to project directly to the OFC subregions we recorded from (VO, VLO, and agranular insular cortex), but its main output structure, the subiculum, does so.^46^^,^^63^^,^^66^^,^^91^ The hippocampus also projects to other structures which in turn target OFC, such as basolateral amygdala,^66^^,^^92^^,^^93^^,^^94^^,^^95^ peri- and entorhinal cortex,^96^ and the thalamic nucleus reuniens.^97^ Moreover, the hippocampal formation emits excitatory projections to the ventral striatum^98^^,^^99^ which in turn controls dopaminergic VTA signaling and ventral pallido-thalamic feedback to prefrontal cortex. Thus, hippocampal output may influence OFC activity through multiple routes. Conversely, the OFC may indirectly affect hippocampal activity via the anterior cingulate, medial agranular, and temporal association cortex.^45^ Given this wide range of indirect connections, the OFC and CA1 likely represent only a subset of a larger network of structures showing coordinated replay.

### Role of orbitofrontal cortex in storing and consolidating task information

When asking whether task-related information is stored in OFC, or whether OFC merely imports information from other areas to compute its outputs on-line,^32^ the present evidence clearly argues in favor of an active role in offline processing (while not excluding information-importing functions in online computations). Because OFC replay is expressed at least by pyramidal cells, it may function to strengthen its internal synaptic matrices or it may affect long-lasting synaptic modifications in downstream areas e.g., striatum, VTA, and basolateral amygdala.^32^^,^^100^^,^^101^ How OFC replay may contribute to the formation of an internal model of task space^22^^,^^23^^,^^24^ is difficult to tell at present. However, one key aspect of model-based learning is the embedding of singular task elements in a spatiotemporal context: subjects learn how associations between cues, actions, and outcomes are contingent on spatial locations and moments within a behavioral sequence.^22^ Thus, the co-reactivation of OFC and CA1 (Figure 6D) may allow the integration of spatial-episodic information coded by hippocampus in a task space model coded by OFC and mPFC to guide flexible and prospective decision-making. Future experiments testing this theoretical framework may flesh out the causal roles of OFC, mPFC, hippocampus, and related structures in these processes.

### Limitations of the study

(1) The current study was based on recordings from three rats. Although this quantity is not unusual in replay studies, this number imposes limitations on the statistical power.
(2) Due to the lack of strong spatial selectivity of orbitofrontal neurons, it was not feasible to reconstruct replayed spatial trajectories from orbitofrontal ensemble activity during rest or task phases.

## STAR★Methods

### Key resources table

**Table:** 

REAGENT or RESOURCE	SOURCE	IDENTIFIER
**Experimental models: Organisms/strains**		

Male Wistar rats, ordered at 7 weeks of age	Envigo	RRID:RGD_5508396
MATLAB	MathWorks	https://www.mathworks.com/
Igor Pro	WaveMetrics	https://www.wavemetrics.com/
Windows Movie Maker	Microsoft	https://www.microsoft.com/
Tool Command Language	N/A	https://www.tcl.tk/
Cheetah	Neuralynx	https://neuralynx.com/

**Other**		

Custom experimental setup	Technology Center, University of Amsterdam	N/A
Custom 14 tetrode hyperdrives	Technology Center, University of Amsterdam	N/A
Nichrome tetrode wire 0.0005 inch diameter Stablohm 800 A (100–189)	California fine wire	CFW2013150
Vibratome	Leica Biosystems	https://www.leicabiosystems.com/

### Resource availability

#### Lead contact

Further information and requests for resources and reagents should be directed to and will be fulfilled by the Lead Contact, Cyriel Pennartz (c.m.a.pennartz@uva.nl).

#### Materials availability

The animal strain used in this study is available from Envigo. This study did not generate new unique reagents.

#### Data and code availability

• All data are available from the lead contact upon request.
• Code, including analysis software, is available from the lead contact upon request.
• Any additional information required to reanalyze the data reported in this paper is available from the lead contact upon request.

### Experimental model and study participant details

#### Animals

Data were collected from three male Wistar rats (Strain: RccHan:Wist), ordered from Harlan Laboratories (the Netherlands) at seven weeks of age. After arrival, the animals were housed in polycarbonate boxes and maintained on a normal day/night cycle (light on at 8:00 h; off at 20:00 h) with food and water available *ad libitum*. Following a habituation period lasting approximately two weeks, rats entered a food restriction schedule with no less than 15 g per rat per day, aimed at maintaining their weight at 80 to 85% of their *ad libitum* weight. To promote reward-seeking behavior during training and mitigate neophobic responses, sucrose solution was administered in the home cage for several days prior to the start of training (15% in water, up to 5 mL per rat daily). All experimental procedures were conducted in accordance with the National Guidelines on Animal Experiments and were approved by the Central Committee on Animal Experiments (CCD) of the Netherlands and the Animal Welfare Committee at the University of Amsterdam.

### Method details

#### Behavioral setup and task

After habituation to housing conditions, rats were trained on the Steering Wheel Maze, a hexagonal maze (main diagonal: 80 cm; Figure 1B) fitted with six equally spaced reward ports delivering a 15% sucrose solution, and with multiple infrared sensors for automated recording of behavioral events.^50^ Two green cue lights were positioned 3 cm above each reward well. The maze was remotely operated using a computer running Tcl custom scripts (Tool Command Language; https://www.tcl.tk) and sensor data were acquired using a Cheetah setup (Neuralynx Inc., USA). During training and testing the maze was weakly illuminated (approximately 50 lux), two visually distinct geometrical cues, located 60 cm away from the maze, were visible at all times, and a speaker providing continuous white background noise was positioned next to the maze in a fixed location throughout all experimental phases.

Animals were initially trained to run clockwise and visit reward ports in a free choice design, with rewards available at all ports except for the recently visited one. On each visit sucrose was delivered if a nose poke was maintained for at least a preset time interval (the minimally required nose poke duration or 'required poke time', taken randomly between 0.25 and 2 s) during cue on (green lights on) periods.

Next, to habituate rats to the varying absence of reward across port visits, they were subjected to an *Equal Probability Schedule*,^50^ where reward probability (p_r_) on all ports was decreased from 100% to 75%. Training on this schedule ended when animals reached a learning criterion of 72 valid trials performed in less than 1 h. A trial was considered valid when nose pokes were maintained at a reward port for at least the duration of the required poke time. This resulted in either sucrose delivery or, for unrewarded trials, activation of an empty syringe pump serving as control for auditory and vibrational cues that would normally occur upon reward delivery.

After successful training on the Equal Probability Schedule, rats transitioned to the flexible Place-Reward Association Task (*fPRAT*, Figures 1A and 1B). Now, rewards were removed from three adjacent ports and *p*_*r*_ was set to 100% on the remaining three ports (Figure 1). Animals received one training session per day and the location of the rewarded ports, relative to extra-maze landmarks, was kept constant for each rat across three training sessions (three consecutive sessions with identical reward distribution made up one *block*, Figure 1B). To minimize the salience of local maze cues (e.g., odor, texture), the Steering Wheel Maze was rotated by at least 60° at the start of each training day while maintaining the position of the rewarded ports relative to the experimental room.^50^^,^^102^^,^^103^ After each block, the position of the rewarded ports was shifted pseudo-randomly by at least two, and at most four, positions (i.e., a rotation by 120°–300°). Rats were trained until they were able to learn the new reward locations and reached a preference of at least 75% for the rewarded ports on the third session of a block. This pre-training phase lasted approximately two weeks, after which four rats were implanted with a tetrode microdrive. Following implantation, animals were subjected to the same *fPRAT* during electrophysiological recordings (Figure 1B). Rats were subjected to either saline or corticosterone (3 mg/kg) intraperitoneal injections using a within-subject design. However, no differences between these two treatment conditions were observed throughout data analysis, therefore these data were pooled.

#### Surgical procedure

Rats were implanted with a custom-built tetrode microdrive featuring two bundles, each containing six individually movable tetrodes and a reference electrode.^104^ Two craniotomies were performed to accommodate the hyperdrive bundles, with the frontal bundle aimed at the lateral orbitofrontal cortex (area LO) and agranular insular cortex (dorsal and ventral part; AID and AIV, Figure 1C; target in left hemisphere: AP: +3.0 mm, ML: −2.6 mm from bregma;^105^ and the caudal one aimed at dorsal hippocampal area CA1 (Figure 1C, right: AP: −4.6 mm, ML: −2.5 mm from bregma). Using dental cement, the microdrive was secured to six surgical screws inserted into the cranium. One caudal screw, situated above the contralateral hemisphere, was used as ground. Over the course of the next ∼9 days the implanted animals were re-trained on the *fPRAT* and the tetrodes were gradually lowered until the regions of interest were reached.

#### Data acquisition

*FPRAT* sessions, flanked by rest episodes lasting between 45 and 60 min, were recorded during the first half of the day (9:00-14:00 h) and rats were subjected to blocks of three sessions until experiments were terminated. OFC tetrode positions were adjusted daily after the recording sessions to optimize the number of recorded units. To ensure recording stability, tetrodes were allowed to stabilize overnight and were never turned in the morning, prior to a recording session.

Maze sensor and electrophysiology data were acquired using a 64-channel Cheetah setup (Neuralynx Inc., USA). Spike channels, band-pass filtered between 600 and 6000 Hz, were sampled at 32 kHz, and data were acquired in a 1 ms window when signal amplitude exceeded a pre-set voltage value. Continuously sampled LFP channels were filtered between 1 and 475 Hz.

Video data, captured via a color CCD camera situated centrally above the maze, were sampled at 30 frames per second with a spatial resolution of 352 x 240 pixels (0.4 cm per pixel) and recorded using Windows Movie Maker software (Microsoft, Redmond, WA, USA). Individual frames were automatically labeled online with numeric codes representing behavioral sensor events (e.g., arm entry) and video data was synchronized offline with sensor and electrophysiological data.

#### Histology

At the conclusion of experiments rats were anesthetized and the end positions of the tetrodes were marked by passing a 12 μA current for 10 s through the tips of the tetrodes. Scar tissue was allowed to form for 24–48 h before the animals were sacrificed with an overdose of Euthasol (80 mg/kg; AST Farma BV, Oudewater, The Netherlands) and perfused with a 4% paraformaldehyde solution in phosphate buffered saline (0.1 M, pH 7.4). After removal from the skull, the brain was stored in 4% paraformaldehyde at 4°C. Serial, 40 μm thick, coronal sections were cut using a vibratome (Leica Biosystems, Nussloch, Germany), and subsequently stained with cresyl violet solution.

Recording locations were estimated by comparing records of daily turning steps and final tetrode positions (Figure 1C, bottom), as determined in stained sections, to a stereotaxic atlas.^105^ Electrophysiological data were obtained from area LO and the ventral (AIV) and dorsal agranular insular (AID) cortices (Figure 1C, left) for the prefrontal bundle, and the CA1 subregion of the dorsal hippocampus for the caudal bundle (Figure 1C, right).

### Quantification and statistical analysis

#### Behavioral analysis

Reward-based spatial learning was measured using a relative place preference index (*RPI*^50^):

(Equation 1) RPI=(N−n)/(N+n)

representing the relative difference between the number of valid trials performed at rewarded (*N*) and unrewarded (*n*) ports.

To measure within-session development of reward site preference, maze run episodes were divided in laps. A lap was defined by two consecutive crossings of the same runway sensor such that, in between, all other runway sensors were activated in clockwise direction at least once. Behavioral analysis was limited to the first eleven laps and the cumulative *RPI*, based on the number of valid trials performed in the current and all previous laps, was calculated for laps 1 to 11 in each *fPRAT* session. Rats performed between 5 and 20 laps per session and sessions with less than 11 laps were discarded. Only the first 11 laps from each session were taken for further analyses. To render performance scores comparable between animals, learning curves from all sessions of each rat were normalized to the maximum attained over the entire session. Unless mentioned otherwise, curves for the first, second and third sessions of each block were averaged across blocks, and mean differences between sessions were calculated across laps. Statistical significance was determined using a Bonferroni-corrected Wilcoxon Matched-Pairs Signed-Rank Test (WMPSRT).

#### Ripples, slow wave activity and Active states

Ripples were detected during quiet wakefulness and non-REM sleep episodes (QW-NREM) in hippocampal LFP data filtered between 105 and 300 Hz (Figure 5A).^29^^,^^36^^,^^40^^,^^106^^,^^107^^,^^108^ All ripples with a squared amplitude that exceeded 4 standard deviations from the mean for at least 6 ms were selected and events shorter than 25 ms, or with an amplitude larger than 2000 μV were excluded from analysis. QW-NREM episodes were detected as time intervals during which rats remained immobile in continuously sliding time-windows of 4 s and the theta band spectral density to overall LPF power ratio did not exceed a value of 0.3.^40^

Periods of enhanced OFC firing activity during QW-NREM, i.e., Active states, were determined from multi-unit data and slow-wave LFP recordings. For a given recording session, spike data from all OFC tetrodes recorded during pre- and post-task rest episodes (*Rest1* and *Rest2*, respectively) were analyzed in 100 ms bins from QW-NREM intervals. Active states were defined as intervals of sustained activity in which the number of spikes exceeded a pre-set threshold defined as 50% of the number of units in that session.^64^ In addition, we determined Up states as a subset of Active states which were preceded or followed by a slow wave within 150 ms.^64^ Slow waves were identified by filtering LFPs between 0.3 and 1.5 Hz, and events above or below 0.5 SD were marked as slow waves.^109^ Slow wave analysis was performed using the YASA Python package.^110^ Additional analysis yielded qualitatively similar results when comparing Up state and Active state properties (similar duration, firing rate and number of spikes per state), with a majority of Active states satisfying the slow-wave criterion used to define Up States (61% of Active states were confirmed as Up states). In the results, however, we mostly refer to Active states and it is important to note that these were not identical to Up states. Note that the number of spikes per Active state showed a graded distribution (Figure S3), indicating that the lowest and highest quartile do not represent discrete classes.

#### Analysis of spike data

A customized version of MClust-3.5 (A.D. Redish) was used for spike sorting. Briefly, using Klustakwik 1.7 (K. Harris), spike data was clustered semi-automatically in multidimensional parameter space, where each dimension was associated with waveform peak amplitudes, energy and the first derivatives of the energy from the four tetrode channels. The resulting spike clusters were refined manually based on spike amplitude stability over time, cross-correlogram properties, and separation quality assessed using the L-ratio and isolation distance.^62^ Clusters with more than 0.1% of inter-spike intervals shorter than the refractory period (1.2 ms and 2 ms for cortical and hippocampal units, respectively), an L-ratio larger than 0.3 and an isolation distance smaller than 15 were excluded.^56^^,^^111^^,^^112^^,^^113^ Subsequently, spike time data and waveforms were imported in MATLAB for further analysis.

Single units from OFC and CA1 were separated into putative pyramidal neurons and interneurons based on the properties of their extracellularly recorded waveforms (Figure S2). For each cluster, spike waveforms were averaged and normalized to the peak amplitude. Subsequently, an agglomerative hierarchical clustering algorithm (clusterdata, MATLAB) was used to discriminate between putative interneurons and pyramidal neurons based on average firing rate and waveform repolarization dynamics, estimated using the waveform amplitude at 0.55 ms after the spike peak.^114^^,^^115^ A large majority of neurons belonged to two cell clusters, one of which exhibited a high firing rate and fast repolarization, and the other showed a low firing rate and slow repolarization. These were classified as fast-spiking, putative interneurons, and putative pyramidal neurons, respectively (Figures 2A, 2B; and S2). Unless otherwise noted, interneurons were excluded from the analyses.

#### Firing rate modulation during non-REM sleep and quiet wakefulness

To assess possible interactions between OFC neuronal activity and hippocampal ripple activity, sharp wave ripple (SWR)-triggered peri-event time histograms (peri-ripple time histograms, PRTHs^57^) were computed from OFC spike data recorded during *Rest1* and *Rest2*. To allow for correct baseline estimation, only peak times from ripples recorded at least 10 s apart were taken. For individual units, ripple modulation was assessed by comparing a time interval from 1 s before to 1 s after ripple peak (0.5 s bin size) to four control bins centered around −5 s using WMPSRT. A bin was considered significantly different from baseline when all four tests reached statistical significance (p < 0.05).

Next, individual PRTHs were z-scored and averages were calculated for both rest episodes. To test whether hippocampal SWRs correlate with overall changes in OFC spiking activity, we used a bootstrapping procedure. For each iteration, we bootstrapped binned firing rate values per OFC cell, recomputed average PRTHs and stored minimum and maximum values. This procedure was repeated 10000 times, and bin values larger than the 95th percentile of the maximum - or smaller than the 5th percentile of the minimum frequency distributions - were considered significant. Statistical differences between mean *Rest1* and *Rest2* PRTHs were computed using a cluster-based permutation test.^116^ Briefly, data was randomized 10000 times and, for each iteration, the value of the largest cluster of datapoints with the t-statistics larger than 1.96 was summed and stored. Using the original data, clusters of data points exceeding the threshold value were compared with the distribution of randomized clustered t-statistics values, and clusters smaller or larger than 2.5 or 97.5 percentile of that distribution were considered significant.

#### Reactivation analysis

To estimate whether temporal activity patterns established during task are preferentially reactivated during post-task rest, we computed Pearson’s correlation coefficients for all pairs of cells recorded in a session. Similarities across the three resulting Pearson’s matrices, corresponding to *Rest1*, *Task* and *Rest2*, were assessed by cross-correlation analysis. Finally, the variance of the correlation pattern observed during post-task rest, explained by novel patterns established by *fPRAT* experience, i.e., after accounting for *Rest1* contributions, was calculated as follows^33^:

(equation 2) $$EV=r_{Task,R1|R2}^{2}={\left(\frac{r_{Task,R2}-r_{Task,R1}r_{R2,R1}}{\sqrt{\left(1-r_{Task,R1}^{2}\right)\left(1-r_{R2,R1}^{2}\right)}}\right)}^{2}$$

where r indicates a matrix-based correlation, calculated for *Task* vs. *Rest1 (R1)*, *Task* vs. *Rest2 (R2)* or *R2* vs. *R1*. The reversed explained variance (*REV*), calculated by switching the temporal order of the rest episodes, served as a control measure for reactivation.^51^ By construction, REV is anti-correlated with EV and therefore these quantities are not independent. However, because EV and REV are temporally mirrored quantities,^51^ they can be directly compared and REV is generally accepted as a control measure for EV. Only sessions with at least six units per brain area were included in this analysis, and control analyses did not reveal increases in firing rates across *Rest1*, *Task* and *Rest2* as reported previously in hippocampus.^55^ Although 20% of units showed at least a 50% increase in firing rate in *Rest2* vs. *Task*, when all cells were included a lower firing rate was observed for OFC during offline states than during Task (Task: 1.36 ± 0.08 Hz vs. Rest1: 1.22 ± 0.07 Hz, p = 0.0016; and vs. Rest2: 1.10 ± 0.06 Hz, p = 5.54e-07, d.f. = 423, Bonferroni-corrected WMPSRT test), with a decrease from Rest1 to Rest2 (p = 0.026, d.f. 423, Bonferroni-corrected WMPSRT test). No such differences were observed for CA1 units (Task: 0.87 ± 0.30 Hz vs. Rest1: 0.71 ± 0.20, p = 1; and vs. Rest2: 0.55 ± 0.17, p = 0.49, Rest1 vs. Rest2: p = 0.17, d.f. = 73, Bonferroni corrected WMPSRT test).

Next, a jackknifing procedure was used to estimate the contribution of each pair of OFC neurons to the overall reactivation per session. Each pair of units was sequentially removed from the dataset and reactivation parameters were recomputed.^56^ The reactivation contribution score (*RCS*) was calculated as:

(Equation 3) $${RCS}_{i}=\frac{{\left(EV-REV\right)-(EV}_{i}-{REV}_{i})}{2}$$

where *EV*_*i*_ and *REV*_*i*_ are the explained and reversed explained variance computed in a given session without pair *i*, while *EV* and *REV* are calculated across all pairs of that session. Subsequently, units (or pairs) were assigned to groups of reactivation *contributors* and *non-contributors* if their corresponding *RCS*_*i*_ fell in the higher or lower 5^th^ percentile of the *RCS* distribution. We did not exclude cell pairs recorded on the same tetrode, as a control analysis using bootstrapped distributions of contributors and non-contributors showed that contributors were less likely to be recorded on the same tetrode than non-contributors.

#### Burstiness of contributor and non-contributor cells

We identified contributor cells as cells which appeared in at least one contributor pair, and did not appear in any non-contributor pair (and the opposite for non-contributor cells). To analyze the potential difference in burstiness between contributor and non-contributor cells, we computed the interspike interval (ISI) distribution (histogram of all inter-spike intervals of a given cell with 5 ms bins between 0 and 200 ms). We then applied a PCA decomposition to the ISI distribution and used a Kolmogorov-Smirnov test to compare the distribution of first and second principal component values between contributor and non-contributor cells.^117^ The first and second principal component explained 87% of the variance in the ISI distribution of all cells (Figure S4).

#### Relationship between behavioral performance and reactivation

We investigated correlations between behavioral performance following changes in place-reward configuration, installed at the onset of each new block, and OFC replay activity during QW-NREM. To this end, we computed the reactivation strength (EV-REV) for each session and its correlation with the change in behavioral performance from the current session to the next. To construct this reactivation-behavioral correlation matrix, only sessions from complete *blocks* (where data from the 1^st^, 2^nd^ and 3^rd^ sessions in a block were available) were selected. Correlations between reactivation strength on *Day d* (with *Day d* corresponding to either session 1 or 2 in a block, Figure 4C) versus the difference in behavioral preference indices (RPIs) between the current session (on *Day d*) and the subsequent session (on *Day d+1*) (corresponding to session 2 or 3, respectively) were computed as follows:

(Equation 4) $$r_{ij}=corr\;\left(\left[{\left(EV-REV\right)}_{d}\right],\left[{RPI}_{d+1,1:j}-\;{RPI}_{d,i:11}\right]\right)$$

where *r*_*ij*_ is Pearson’s correlation coefficient between the reactivation strength ${\left(EV-REV\right)}_{d}$ (where *d* represents the session rank in a block and assumes a value of 1 or 2) and the behavioral performance difference, which is the difference between the *RPI* on *Day d+1* (*RPI*_*d+1,1:j*_), computed over laps 1 to *j* (with *j* from 2 to 11), minus the *RPI* on *Day d* (*RPI*_*d,i:11*_), computed over laps *i* to 11 (with *i* from 1 to 10). This analysis was performed over all consecutive sessions in a block and resulted in a 10 × 10 correlation matrix with elements *r*_*ij*_. In other words, *r*ij (e.g., r_6,2_ for lap 6 on *Day d+1* and lap 2 on *Day d*) represents the correlation coefficient between two sets of values, (1) the reactivation strengths for all *Day d* sessions (that is, sessions 1 and 2 from all blocks) versus (2) all differences between the RPI values for *Day d+1* and *Day d* sessions, with the RPI for *Day d+1* in this example calculated for laps 1 to 6 and the RPI for *Day d* calculated for laps 2 to 11 (Figure 4C2).

Pearson’s correlation coefficients were evaluated for significance using a bootstrapping procedure. Data were randomly sampled with replacement 10000 times to obtain, for each iteration, a new dataset where lap identity was randomized. Here, we exclusively used contiguous lap combinations presented in the reactivation-behavioral correlation matrix (i.e., without discontinuous subsets of laps). Peak correlations from each bootstrapped correlation matrix were used to build a probability distribution (Figure 4C3), and *r*_*ij*_ values were considered significant if they exceeded the 95^th^ percentile of this distribution.^116^^,^^118^

#### Analysis of temporal bias in cross-correlations

Specific temporal relationships between neuronal pairs were evaluated as previously described.^40^^,^^54^ Cross-correlograms were calculated between all neuronal pairs in each recording session for all three task episodes (*Rest1, Task, Rest2*) and integrals of the cross-correlograms were computed across 400 ms before and after zero. To obtain a normalized measure of temporal bias (*t*_*B*_), their difference was divided by the integral over an 800 ms window centered on zero:

(Equation 5) $$t_{B}^{ij}=\frac{\int_{0}^{400}r_{ij}\left(t\right)dt-\int_{-400}^{0}r_{ij}\left(t\right)dt}{\int_{-400}^{400}r_{ij}\left(t\right)dt}$$

where $t_{B}^{ij}$ is the temporal bias value of cells i and j and $r_{ij}$ is the crosscorrelation of the binned firing rates of cell pair *ij*, with *i* and *j* being single units from the same session.

We evaluated the differences in Pearson’s correlation coefficients, calculated for *t*_*B*_ during *Rest1* vs. *Task* and *Rest2* vs. *Task*, using bootstrap statistics. Temporal bias datasets from both rest episodes were bootstrapped (i.e., episode identity was shuffled) and their correlations to *Task t*_*B*_ values were recomputed. This step was repeated 10000 times and correlation differences were compared against the lower and upper 2.5 percentiles of the bootstrapped probability distribution. This procedure was applied to test the statistical significance of datasets from both contributor and non-contributor pairs of OFC units.

#### Rate-map correlations

Rat head position data were obtained from video files, using a brightness threshold-based algorithm, which identified Cartesian coordinates of microdrive-mounted LEDs. To construct firing rate maps, data were binned into 5 × 5 pixels and occupancy-corrected firing rates were calculated for each cell and each spatial bin for the entire duration of the *Task* episode. Matrix-based correlation coefficients between the rate maps of all neuronal pairs were calculated using a MATLAB function (corr2) and the statistical difference between contributor and non-contributor units was evaluated with a Wilcoxon Rank-Sum Test (WRST).

#### Spatial firing parameters

Spatial tuning of hippocampal and OFC single units was assessed using two parameters. First, spatial information content was calculated as:

(Equation 6) $$I=\sum_{i=1}^{N}p_{i}\frac{\lambda_{i}}{\lambda }\log_{2}\frac{\lambda_{i}}{\lambda }$$

where *I* is the spatial information content calculated for a single neuron, *p*_*i*_ is the probability of the rat occupying spatial bin *i,* *λ*_*i*_ and *λ* are the mean firing rate in bin i and the average firing rate across all *N* bins, respectively.^119^^,^^120^ The second parameter, spatial selectivity, was computed by dividing the maximal firing rate by the mean firing rate across bins.^107^^,^^121^

Unless otherwise specified, all analyses were performed using custom-written MATLAB scripts and data are shown as median or mean ± standard errors of the mean (SEM).
