# Supplementary material for: Offline orbitofrontal cortex reactivation depends on recency of place-reward changes and coheres with hippocampal replay
Source: iScience. 2024 Feb 10;27(3):109205. doi: 10.1016/j.isci.2024.109205 (PMC10933476; doi:10.1016/j.isci.2024.109205)
Supplement: Document S1. Figures S1–S4 and Table S1 [file mmc1.pdf]

iScience, Volume *27*

Supplemental information

Offline orbitofrontal cortex reactivation depends on recency of place-reward changes and coheres with hippocampal replay

Silviu I. Rusu, Jeroen J. Bos, Pietro Marchesi, Jan V. Lankelma, Ildefonso Ferreira Pica, Luc J. Gentet, Marian Joëls, and Cyriel Pennartz

Figure S1

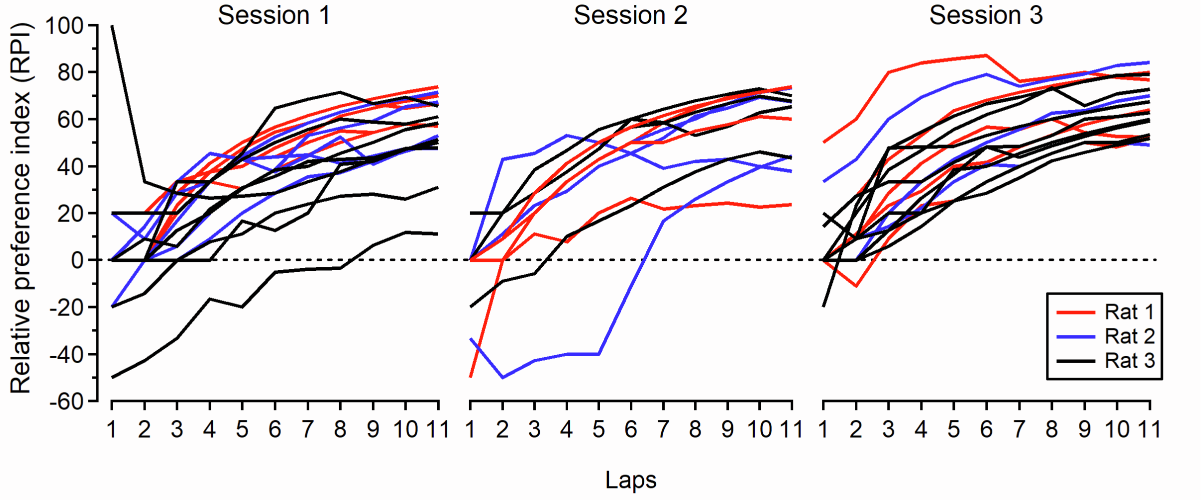

Figure S1. Behavioral performance of individual rats in individual sessions, related to Figure 1. Non-normalized cumulative relative place-preference index (RPI) calculated as the relative difference between the number of valid trials (see Methods) at rewarded and unrewarded ports for each lap and preceding ones. RPI learning curves from all sessions with at least 11 laps per session are grouped by rank within a block (session 1 to 3 from left to right) and pooled across all blocks and rats. Note the overall higher RPI at the session start and the increased consistency in performance in session 3.

Figure S2

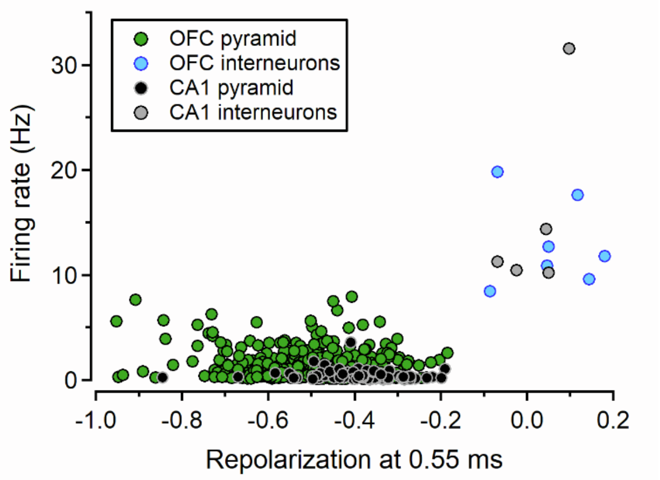

Figure S2. Putative OFC and CA1 pyramidal cells and interneurons, related to Figure 2**.** Classification of OFC and CA1 into putative pyramidal cells and fast-spiking interneurons was done in a two-dimensional space defined by firing rate and repolarization amplitude at 0.55 ms from the peak of the mean normalized waveform, taken from the tetrode lead with the highest amplitude.

Figure S3

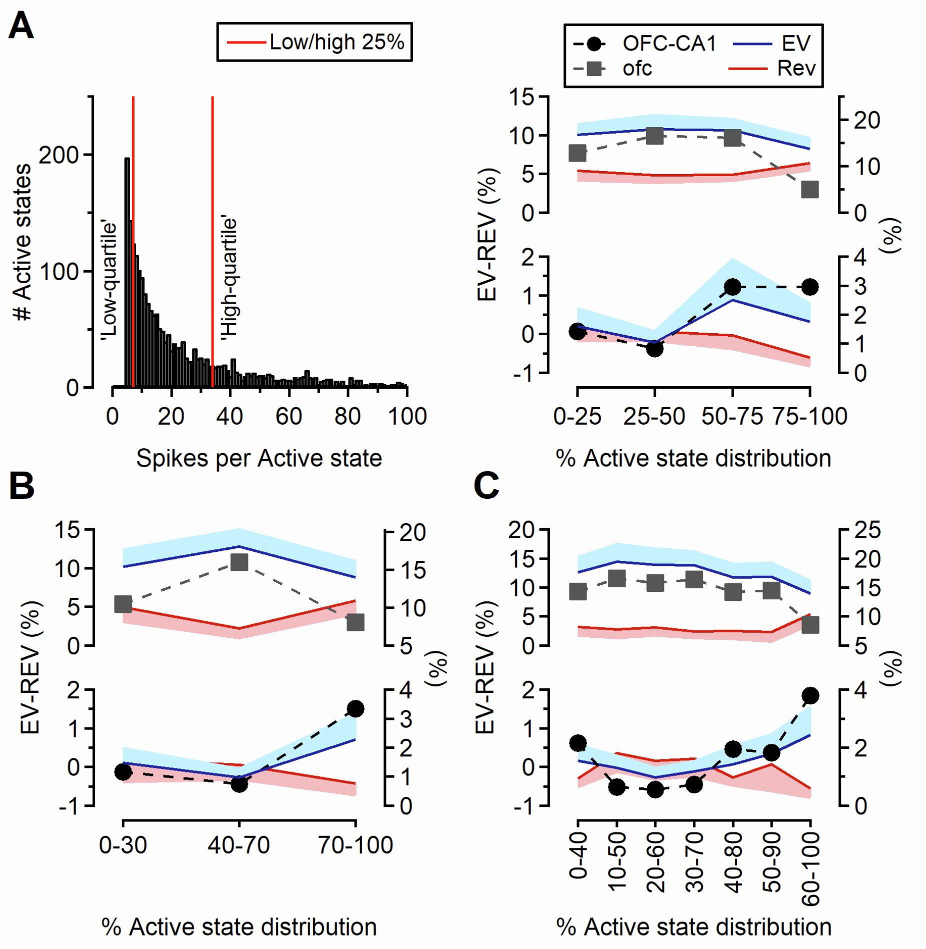

Figure S3. Orbitofrontal cortex and Orbitofrontal cortex-CA1 reactivation strength across different percentile ranges of Active state, related to Figure 6**.** A, left: Distribution of Active states calculated for the number of spikes per Active state during offline states from an example session. Red vertical lines indicate 25th and 75th percentiles of the distribution, Active states falling below and above these thresholds were termed Sparsely (low-quartile) and Highly (high-quartile) Active states, respectively. A, Right: Average OFC and CA1 reactivation strengths calculated across sessions for each of the four quartiles of the Active states distribution exemplified in A, left. Here, reactivation strengths calculated for the top and bottom Active state distribution quartiles correspond to the data presented in Fig. 6C, D (6C: intra-OFC; 6D: OFC-HPC correlations). Blue and red lines show mean EV and REV, respectively, calculated for all percentile ranges (SEM: shaded areas). B: same as A, except that reactivation strengths were computed for the bottom, middle and top 30% of the data. C: same as A, except the reactivation strength was calculated for a 40 % moving window, in 10% increments. Note that the reactivation strength profile across different Active state distribution percentiles is largely conserved across different percentile intervals, with the OFC showing larger and smaller EV-REV differences at the left and right of the Active state distribution, respectively, and OFC-CA1 reactivation displaying the opposite pattern.

Figure S4

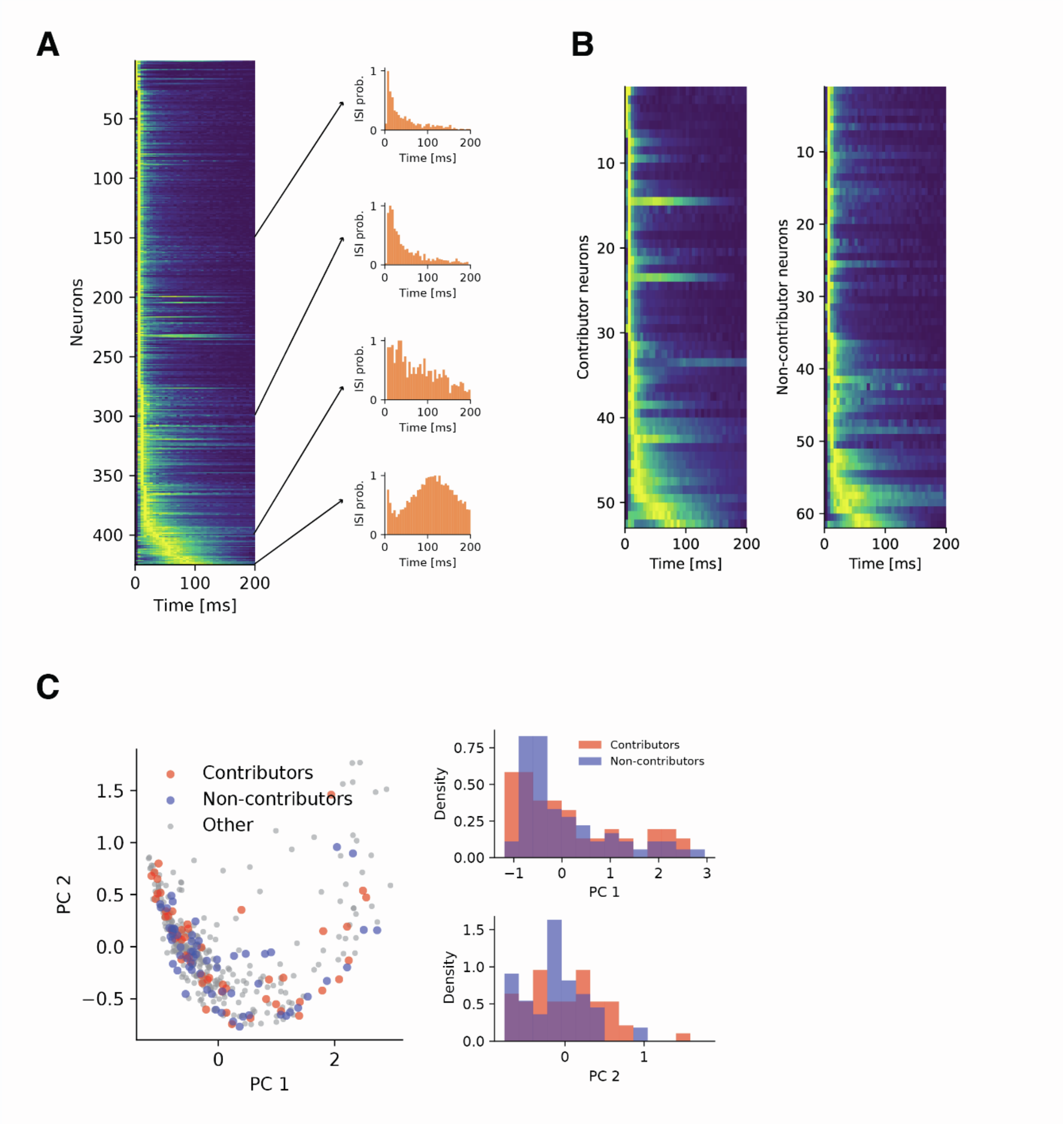

Figure S4. **Burstiness of contributor and non-contributor neurons in orbitofrontal cortex,** related to Figure 3. A, Left: Histogram of the inter-spike intervals (ISIs) of all cells. Right: normalized histograms of the ISIs of individual cells with different burstiness profiles. Burstiness increases towards the bottom of the graph. B, Histograms of the ISIs of contributor cells (cells which appear in at least one contributor pair, and do not appear in any non-contributor pair, left) and non-contributor cells (cells which appear in at least one non-contributor pair, and do not appear in any contributor pair C, Left: PCA decomposition of the ISI histograms for contributor (red), non-contributor (blue), and other cells (grey). The first and second principal component explained 87% of the variance in the ISI distribution of all cells. Right: histograms of the first principal component values (PC1, top) and second principal component values (PC2, bottom) for contributor (red) and non-contributor cells (blue). Kolmogorov–Smirnov tests indicated no significant difference between contributor and non-contributor distributions of PC1 and PC2 values.

Table S1. Overview of analysed sessions, related to Figure 2**.** For each rat the recorded sessions (Rec) in chronological recording order, the number of units per session and the number of reward switches per rat are listed. Analyses of OFC and CA1 activity were restricted to sessions with at least 6 units per area and per session, yielding 10 and 36 sessions for CA1 and OFC analyses, respectively.

Rats	Recording session	n OFC units	n CA1 units	n reward switches
**Rat1**	Rec01	8		3
	Rec02	9		
	Rec03	12		
	Rec04	7		
**Rat2**	Rec01	17		6
	Rec02	15		
	Rec03	6	6	
	Rec04	12	7	
	Rec05	13	7	
	Rec06	12	6	
	Rec07	8		
	Rec08	10	6	
	Rec09	14	9	
	Rec10	7	6	
	Rec11	8		
	Rec12	6		
	Rec13	16		
**Rat3**	Rec01	10		7
	Rec02	7		
	Rec03	10		
	Rec04	10		
	Rec05	6		
	Rec06	13	9	
	Rec07	15		
	Rec08	22		
	Rec09	13		
	Rec10	12		
	Rec11	21		
	Rec12	22		
	Rec13	15		
	Rec14	16		
	Rec15	7		
	Rec16	11	10	
	Rec17	12		
	Rec18	11	8	
	Rec19	11		
